# Experimental and FE Study on Impact Strength of Toughened Glass–Retrospective Approach

**DOI:** 10.3390/ma14247658

**Published:** 2021-12-12

**Authors:** Marcin Kozłowski, Kinga Zemła, Magda Kosmal, Ołeksij Kopyłow

**Affiliations:** 1 Department of Structural Engineering, Silesian University of Technology, Akademicka 5, 44-100 Gliwice, Poland; 2 Faculty of Civil Engineering, Silesian University of Technology, Akademicka 5, 44-100 Gliwice, Poland; kingzem817@student.polsl.pl; 3 Łukasiewicz Research Network Institute of Ceramics and Building Materials, Cementowa 8, 31-983 Krakow, Poland; magda.kosmal@icimb.lukasiewicz.gov.pl; 4 Building Research Institute ITB, Filtrowa 1, 00-611 Warsaw, Poland; o.kopylov@itb.pl

**Keywords:** experiments, finite element, glass, impact, toughened glass

## Abstract

Due to the high cost of experiments commonly performed to verify the resistance of glass elements to impact loads, numerical models are used as an alternative to physical testing. In these, accurate material parameters are crucial for a realistic prediction of the behaviour of glass panels subjected to impact loads. This applies in particular to the glass’s strength, which is strictly dependent on the strain rate. The article reports the results of an extensive experimental campaign, in which 185 simply supported toughened glass samples were subjected to hard-body impacts. The study covers a wide range of glass thicknesses (from 5 to 15 mm), and it aims to determine a critical drop height causing fracture of the glass. Moreover, a 3D numerical model of the experimental set-up was developed to reproduce the experiments numerically and retrospectively to determine the peak stress in glass that developed during the impact. Based on the results of numerical simulations, a load duration factor of 1.40 for toughened glass for impact loads is proposed. In addition, the paper includes a case study to demonstrate the use of the modelling methodology and results of the work on a practical example of an internal glass partition wall.

## 1. Introduction and Motivation

The application of glass in buildings has increased expeditiously over the past decades [1]. This is driven by its intrinsic characteristics such as transparency, alluring appearance, high strength and resistance to environmental factors [2]. This trend particularly applies to canopies, building enclosures and load-bearing structural elements inside buildings [3]. Besides bearing typical (static) loads, these elements may be exposed to exceptional loads during their lifetimes, such as blast, fire, and impact loads [4,5,6].

The elements are commonly made of fully toughened (FT) glass, which due to the residual stress developed in a tempering process, shows increased strength, impact and high-temperature resistance compared to ordinary annealed glass [1,7,8]. All glass elements made of FT glass should meet the basic requirements as all construction products in the European Union according to the directive Construction Products Regulation [9]. Its physical and mechanical characteristics are specified by standards EN 12150-1 [10] and EN 12150-2 [11]. To meet these specifications and fulfil the codes’ requirements, standard tests are performed. 

The fragmentation test defines the safety level and gives an indication about the stress level in FT glass panes [12,13]. The test is conducted on glass samples with dimensions of 360 × 1100 mm^2^ according to EN 12150-1 [10]. The samples are impacted with a special tool at the midpoint of the longest edge. An edge strip (25 mm in width) and the area inside a circle (100 mm radius) around the point of impact are disregarded for assessing the fragmentation. The evaluation consists in counting the fractured glass fragments inside a 50 mm square and measuring the length of the elongated fragments. The number of counted fragments may not be less than the required in the standard, and the length of any elongated fragments may not exceed 100 mm. To meet the requirements, each of the tested samples must obtain a positive result. 

Resistance of FT glass to soft-body impact is assessed according to EN-12600 [14]. The test is conducted in accordance with the methodology described in the standard on samples with dimensions of 876 × 1938 mm^2^. The two-tire, 50 kg pendulum impacts the samples installed in a test frame from three drop heights depending on the load class (190, 450 and 1200 mm). It is allowed to use samples that did not break when impacted from a lower height. 

It should be emphasised that the aforementioned tests are limited to a qualitative assessment of the performance characteristics and do not provide quantitative information about the impact strength of the material, which is crucial in design. 

Determination of the bending strength (under static loads) of FT glass is performed in accordance with EN 1288-3 [15]. A four-point bending test is performed on glass samples with dimensions of 360 × 1100 mm^2^. The samples are placed on a support with two rubber-lined metal rollers, 50 mm in diameter, 1000 mm apart. The samples are loaded with a rate of 2 ± 0.4 MPa/s until breakage. The bending strength of the glass is calculated from the value of braking force. In addition to the load, deflection and the duration of bending to failure are noted. The strength of tested FT glass samples should not be less than 120 MPa.

In comparison to the fragmentation test and the soft-body impact (which provide purely qualitative data), the bending strength gives a specific number that can be used further in design. However, it applies only to the static strength of glass. It should also be noted that standard tests are performed on the clearly defined samples with the dimensions adapted to tests on a laboratory scale and the results of these experiments cannot be directly translated to life-size samples [1]. Therefore, it is important to create such a research platform that allows verifying the obtained laboratory results on real-scale samples used in construction. 

Design procedures for dimensioning glass elements for static loads are well known and standardised [16]; however, it is different for dynamic loads, which in most cases, govern the final thickness of glass [17,18]. Nowadays, verifying the load-bearing capacity of glass elements to dynamic loads is conducted by laboratory testing of real-scale elements with dimensions and fixing methods corresponding to on-site details [19,20]. However, these types of tests are time-consuming and costly. Moreover, the methodology does not consider the real on-site conditions because the tests are carried out in a laboratory under controlled conditions (constant temperature, humidity, etc.). Furthermore, when taking into account the significant scatter of the glass strength obtained from the material research [1,7], the results of a single laboratory test should not be treated as comprehensive data, and it does not confirm the designed load-bearing capacity of an element and its structural safety.

Numerical models are an alternative to expensive and time-consuming laboratory tests. They allow for a comprehensive analysis of various design cases, taking into account variable material properties and different load combinations (static, dynamic etc.), geometry and design situations [4,21,22,23,24,25]. Numerical modelling allows for an in-depth analysis of the dynamic response of the structural components in opposition to simplified models [26,27], which, due to the needed assumptions and simplifications, mostly result in oversizing the glass thickness. Moreover, the glass standards currently under development allow the use of validated numerical methods for designing structural elements made of glass [28]. 

Regarding the numerical models, accurate material parameters are crucial for a realistic prediction of the behaviour of a glass panel subjected to an impact load [29]. This applies particularly to the strength of glass, whose strain rate dependency is well known in structural glass design [30,31,32]. Experimental research confirms an increase in bending strength of glass under short-term loads [29,33,34,35,36,37]; however, most of them, due to the limited number of tested samples, do not allow for a verified assessment of glass strength under impact loads. 

The strain rate dependency was implemented differently in standards [16,38,39]. In the European standard EN 16612 [16], the load duration factor *k_mod_* considers the rate dependency, affecting the strength component related to annealed glass. For exceptional loads of very short duration, the value of *k_mod_* may be greater than 1.0 and cannot be calculated according to the provided formula, because it is limited only to dynamic events with a time duration longer than or equal to 20 ms. In opposition to the European approach, the German standards DIN 18008-1 [38] and DIN 18008-4 [39] refer to each different glass type and provide load duration factors *k_mod_* only for soft-body impacts, which duration is rarely shorter than 100 ms [40,41,42]. As shown in the literature [20,37,43,44,45], a hard-body impact lasts much shorter (less than 1 ms) in comparison to the soft-body impact. Therefore, an adequate value of the load duration factor should be proposed. 

The current work is a part of the ongoing research project focused on an innovative solution for point-fixed laminated glass with improved capacity after glass fracture. It continues our reported exploratory research [46] and involves a significantly larger number of samples and improved research methodology. It contributes to the existing knowledge by proposing a load duration factor for FT glass subjected to hard-body impact. Firstly, it reports the results of an experimental campaign that includes testing glass samples with thicknesses commonly used in building engineering (from 5 to 15 mm) under hard-body impact. To obtain statistically reliable data, 185 glass specimens were tested. Secondly, based on the results of the experiments, statistical evaluation is performed to obtain critical parameters for a further numerical study aimed at retrospective determination of glass strength under impact loads. Subsequently, a load duration factor for FT glass subjected to impact loads is proposed on the basis of Eurocode regulations and compared to another research. Finally, a case study is presented to demonstrate the potential of the modelling methodology and provide an example of using the results of this work in practice.

## 2. Materials and Methods

### 2.1. Materials

The study decided to use regular soda-lime silicate float glass, which is the most popular type of glass used in the civil engineering industry and for architecture applications. The study focuses on FT glass due to its higher strength than ordinary annealed glass [1,7]. During the thermal tempering process, in which all FT glass panes are submitted, prestress compressive zones occur, increasing the glass’s strength. In the cross-section of the sample made of FT glass, compressive stresses appear on the outer surface, whereas on the inner part, tensile stresses form, which are in balance [1,7]. As a result, the mechanical strength of the FT glass is over two or three times higher than the strength of the annealed float glass [1,47]. The characteristic tensile bending strength of FT glass equals 120 MPa [10], whereas, for annealed glass, this value is significantly lower, equals to 45 MPa [48].

### 2.2. Experimental Testing

The section presents the results of an experimental campaign focused on the destructive energy caused by the free fall of a steel ball and required to fracture glass samples. The experimental campaign was built upon the results collected by Ziemba et al. [49]. The tests were originally published in an internal, technical report of the Institute of Ceramics and Building Materials (Krakow, Poland) [49]. The tests were devoted to determining the critical destructive height for the drop ball test, in which a steel ball is fallen on the glass sample. The report was key to determining the maximum height at which the glass samples were fractured for different thicknesses of the panes. The main issue of the study is the impact resistance of glass panes which is related to destructive energy strictly correlated with the critical height determined by Ziemba et al. [49]. This is a relevant topic in designing structural elements made of glass since, in most cases, the impact loads govern the final thickness of glass elements [17,18].

In the experimental campaign, panes with dimensions of 500 × 360 mm^2^ were used. The samples were made of FT monolithic glass with various (nominal) thicknesses: 5, 6, 8, 10, 12 and 15 mm. It was essential to use samples with polished edges because the strength of the glass pane is strictly connected with the level of the edge’s finishing. For polished edges, the possibility of the appearance of the Griffith flaws is considerably smaller than seamed or grind edges [50]. Thus, the strength of samples with polished edges is greater than the other types.

In general, the bending test results show significant variability of glass strength because of the microcracks on the surface of the panes, which lowers the load-bearing capacity of the sample. Therefore, it was assumed to utilize a large enough number of samples that is sufficient for statistical analysis and for precise calculation of the bending strength of the samples in the next steps. The standard related to the subject [51] states that the number of the samples should be large due to uncertainty and common large scatter of the results. Therefore, the experimental campaign was performed for the 185 samples, 35 samples for each series. In series with 5 mm glass thickness, the number of samples was equal to 10. 

**Figure 1:**
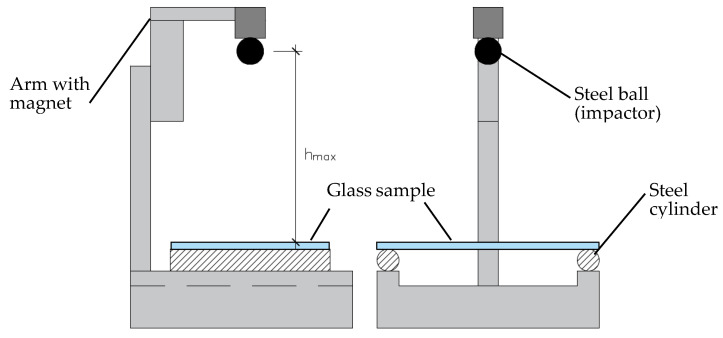
Custom-build machine used in the testing. Side- and front views of the machine.

The tests were performed using a custom-built machine, which is shown in Figure 1. The machine consisted of steel cylinders with 50 mm in diameter and 365 mm in length and an electromechanical arm with a magnet. To avoid accidental stresses at the support zones, samples were placed on the steel cylinders, covered with a thin layer of elastic rubber material. A steel ball (impactor) was placed on the arm with the magnet, and the initial height was measured- equalled to 10 cm over the sample. The impactor used in the experimental campaign had a weight of 4.11 kg. Tests were conducted to achieve a critical height above the sample, from which dropped steel ball fractured the specimen. The increment of the height was equal to 10 cm. The tests were performed at the relative humidity of 50% and at room temperature.

### 2.3. Numerical Modelling

The main aim of the finite element (FE) study was to numerically reproduce the performed experiments and evaluate the history of impact force and principal (tensile) stress in the glass. Based on the maximum value of the obtained stress, a characteristic value of the glass strength was evaluated. To do so, a three-dimensional numerical model of the experimental set-up (Figure 1) was developed using the commercial FE analysis software SIMULIA ABAQUS 2020 (Dassault Systèmes, Vélizy-Villacoublay, France) [52]. A quarter of the nominal geometry of the set-up together with adequate boundary conditions was modelled to reduce the number of FEs and increase the computational efficiency of the simulations. All simulations were run in the Implicit Dynamic solver [53,54].

**Figure 2:**
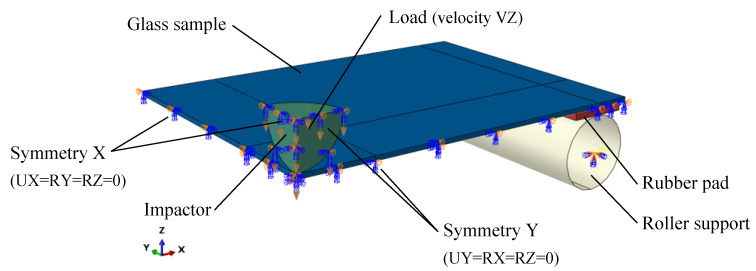
General view of FE numerical model (ABAQUS/Standard).

A numerical reference model is shown in Figure 2. It consists of a glass sample, an impactor, a rubber pad, and roller support. The glass sample was modelled with a set of 3D 8-node, solid elements with full integration (C3D8 type from ABAQUS element library [52]). In the study, different thicknesses of the samples were considered, which corresponded to the nominal thicknesses of the glass panes used in the experimental campaign. The impactor was modelled with 3D 8-node, solid elements with full integration (C3D8 type from ABAQUS element library [52]). The roller support was modelled using a discrete rigid surface (R3D4 type from the ABAQUS element library [52]). The rigid surface corresponds to the outer surface of the roller support used in the experimental campaign. The rigid surface was tied to a reference point with fully fixed boundary restraints applied. Between the glass sample and the roller support, a rubber pad was modelled with 3D 8-node, solid elements with full integration (C3D8 type from ABAQUS element library [52]). Between the sample and the impactor and between the sample and the rubber pads, surface-to-surface contact interactions that allowed lifting and relative sliding with a friction coefficient of 0.7 were assumed [55]. Regarding the impactor, translation along the Z (vertical) axis was released to allow the impactor to be set in motion. 

The glass was represented using linear elastic material properties with the density ρ = 2500 kg/m^3^, Young’s modulus E = 70 GPa and Poisson’s ratio ν = 0.23 [16]. Even though monolithic glass damping effects are known to be limited [20], a value of viscous damping ratio was set to ξ = 1% [22,56]. The damping of the glass panes was simulated using Rayleigh α- and β-damping, which correspond to the mass (α) and stiffness (β) proportional damping. The derivation of the Rayleigh parameters was performed based on two first natural periods (T_1_ and T_2_) of simply-supported glass panels [57]. The prescribed values of Rayleigh parameters (α and β) for different glass thicknesses are presented in Table 1. The steel ball was modelled using linear elastic with the density ρ = 7850 kg/m^3^, Young’s modulus E= 200 GPa, and Poisson’s ratio ν = 0.3 [58]. Due to the modelling approach, in which only one-eighth of the impactor was modelled, the density of steel was doubled. The rubber pad was modelled with the following linear elastic material model properties: Young’s modulus of 100 MPa (10 × typical value for hard rubber materials) and ν = 0.48 [59]. To account for the high damping characteristics of rubber, the value of the viscous damping ratio was set to ξ = 5% [60]. 

An irregular mesh pattern was applied to the glass pane. At the impact location (zone 50 × 50 mm^2^), a fine mesh was applied, while for the other zones of the sample, a coarse mesh corresponding to half of the thickness of the sample was used. In the thickness of the pane, three FEs were defined. In terms of the impactor, the same mesh approach was used. For the rubber pad and the discreet rigid surface (roller support), the mesh size was equal to the mesh pattern of adjacent components. To simulate the collision of the impactor and the specimen, the impactor was set into motion with an initial velocity further detailed in Section 3.2.2.

## 3. Results and Discussion

### 3.1. Experiments

The experimental campaign was key to determining the destructive energy (represented by kinetic energy) which fractures the samples. In the report [49] only critical drop height was measured, which is not a proper result for the numerical analysis presented in the next section. The kinetic energy was calculated, which is strictly related to the maximal height of the drop. Using the value of the destructive energy, it was possible to recreate experiments in future works, with other weights of the impactor and critical height. Furthermore, in the numerical analysis, the impactor’s velocity should be modelled; thus, critical height is not the leading factor. To determine the kinetic energy, it was vital to beforehand determine the velocity of the impactor. The ball’s motion was assumed as a free fall from a given height; thus, the impactor’s velocity could be presented as the following Equation (1):

(1) $$v_{max}=\sqrt{2gh_{max}}$$

It was assumed that gravitational acceleration (g) equals 9.81 m/s^2^ and critical drop height (*h* = *h_max_*) is given in meters. In the next step, the kinetic energy was determined with a received value of the velocity (Equation (2)):

(2) $$E_{k}=\frac{1}{2}mv_{max}{}^{2}$$

It was significant to use the characteristic value of the obtained results. In the next steps, the characteristic value of the bending strength of the samples was determined to further compare them with the value proposed in the standards. 

According to the literature on the subject related to statistical evaluation of results obtained from experimental testing [61,62], it is necessary to determine the characteristic value of the tested factor. It was key to assume that log-normal distribution could be applied because, in the experimental investigation, only positive values were obtained. Firstly, a velocity for each critical height for every thickness was calculated, and upon these values, kinetic energy was determined according to the abovementioned equations (Equations (1) and (2)). For the log-normal distribution, the factor’s lower characteristic value (5% fractile) should be determined as stated in Equation (3) [62]:

(3) $$X_{k}=\exp\left[m_{y}-k_{n}s_{y}\right]$$

where *m_y_* is the mean value of the natural logarithm of each value. In this case, it is the mean value of the natural logarithm of kinetic energy, whereas *S_y_* is the standard deviation calculated as follows (for a known value of *Vx*):

(4) $$s_{y}=\sqrt{\ln\left(V_{x}{}^{2}+1\right)\approx V_{x}}$$

It was vital to determine the specific value of *k_n_* factor, which is a characteristic fractile factor. There are only given values of the factor with a given number of samples and known or unknown value of *Vx* in the standards. Unfortunately, the function is not linear; thus, it is impractical to interpolate values not specified in the standard (for *n* = 35). Thus, it was decided to follow the method proposed in the standard, but the value of the characteristic fractile factor was calculated as proposed by Bond et al. [63].

For case: statistical “known” parameter and extreme value, *k_n_* factor is expressed as follows, assuming that there is a 95% level of confidence:

(5) $$k_{n}=t_{\infty }{}^{95\%}\sqrt{\frac{1}{n}+1}$$

In Equation (5), the value of *t* in the infinity with the confidence level equals 95% for infinite degrees of freedom, taken from Student *t*-distribution is approximately equal to 1.645 [63]. In the above-mentioned equation, *n* is equal to the number of samples. Thus, for the number of samples (*n* = 35) *k_n_* factor equals 1.668. Whereas, for the series with ten samples, the *k_n_* factor is equal to 1.725. This value is similar to those given in the standard [62] from the estimated analysis. 

Table 2 presents results from the experimental campaign devoted to impact tests with calculated kinetic energy values required to destroy the sample. Mean values of destructive height with corresponding values of standard deviation and equivalent kinetic energy were presented. In the table, the characteristic kinetic energy is given. Although only ten specimens were tested with the thickness of 5 mm, the factor (*k_n_*) differs only 3.5% in comparison to 35 samples, thus the number of samples (ten) is considered sufficient for the analysis.

**Figure 3:**
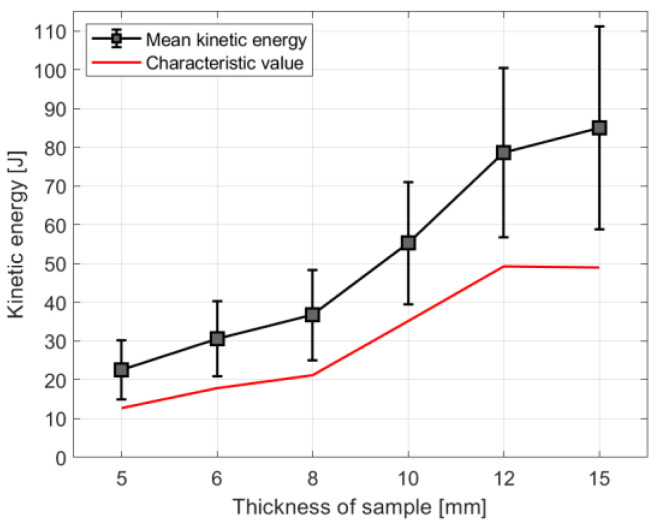
Diagram of kinetic energy vs thickness of the sample.

Figure 3 shows the relationship between the values of kinetic energy and the thickness of the sample. It is evident that for thicker samples, larger kinetic energy is necessary to fracture the samples, thus, with increased thickness, the kinetic energy increases. The analysis shows that to break a thicker sample, higher impact energy is needed. In the literature [64], the same observation was made. Moreover, with increasing the sample’s thickness, the standard deviation of received results increases (Figure 3).

It was vital to mark in Figure 3 the characteristic values of the kinetic energy to present the relationship between them and results obtained in the experimental campaign. The characteristic values of kinetic energy should be used in numerical analysis. It is possible to notice that characteristic values are lower than the lower limit of the standard deviation of each result from experimental tests. Furthermore, the difference between the results from the experiment and the characteristic value is greater for the results with a larger standard deviation.

### 3.2. Numerical Study

#### 3.2.1. Convergence Study

To validate a reference FE model, a mesh convergence study was performed. The procedure aimed to verify the mesh quality and investigate how the reference model (5 mm in thickness) converges to the true solution depending on the FE size of the glass sample.

**Figure 4:**
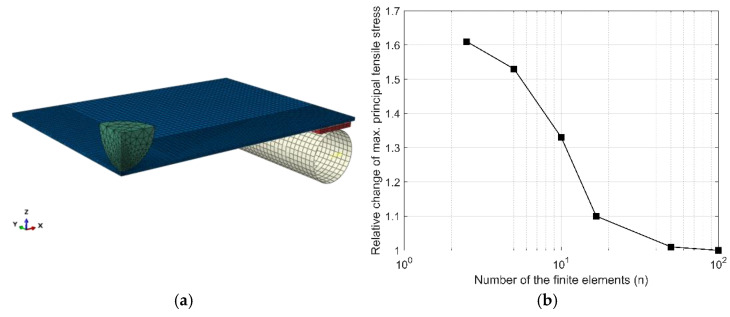
Mesh convergence study: (**a**) final FE mesh used in further study, (**b**) relative change of output variable extrema versus the number of FEs along the impact zone.

In the study, a FE mesh presented in Figure 4a was analysed. The FE mesh of the zone at the impact location (50 × 50 mm^2^) was varied by reducing the number of FEs along the edge of the zone. The results of the convergence studies were analysed by examining the relative change in the output variable extrema–in this case, the maximum principal (tensile) stress in the glass. 

Figure 4b shows the relationship of the relative change (in relation to the previous step) of the maximum principle (tensile) stress in glass to the number of FEs along the impact zone (n). Based on the results of the convergence study, it was concluded that the model with 50 FEs along the impact zone edge converges to a sufficient degree (the relative change of the output variable, in relation to the maximum value with 100 FEs, is less than 1%). This FE mesh pattern was kept throughout all further analyses and resulted in 20,700 FEs and 95,700 degrees of freedom.

#### 3.2.2. Retrospective Evaluation of Glass Strength

To simulate the collision of the steel ball and the specimen, the impactor was set into motion with a velocity *v_k_* calculated according to Equation (6). The equation results from the transformation of a formula for kinetic energy with the assumption that for the considered case, the kinetic energy equals the potential energy of the impactor before setting into motion [18,41]. Equation (6) allows calculating the initial velocity *v_k_* (in meters per second) for a given value of the characteristic kinetic energy *E_k,k_* determined in the experimental campaign (Table 2) and the mass of the impactor *m*. The values used in the numerical analyses are shown in Table 3.

(6) $$v_{k}=\sqrt{\frac{E_{k,k}}{0.5\times m}}$$

**Figure 5:**
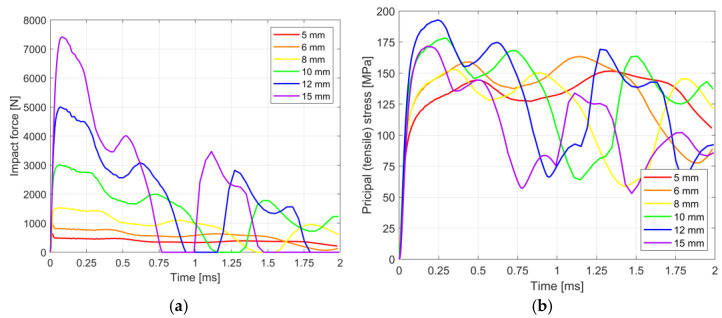
Results of numerical studies. History of: (**a**) impact force, (**b**) principal (tensile) stress in glass at the impact location.

Figure 5a,b present the history of impact force and the corresponding principal tensile stress in glass at the impact location in a time smaller than 2 ms of the dynamic analysis. The plots were obtained from numerical analyses for the glass samples with different thicknesses and given the characteristic kinetic energy values determined in the experimental campaign (Table 2). 

**Figure 6:**
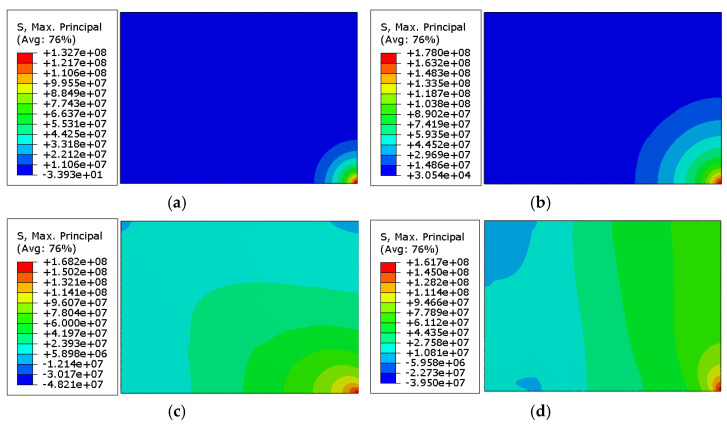
Results of numerical studies. Principal stress maps for 10 mm specimen at times: (**a**) 0.06 ms, (**b**) 0.29 ms, (**c**) 0.73 ms, (**d**) 1.51 ms. Values in the legend are shown in Pa.

It can be noticed from Figure 5a,b that the hard-body impact is an extremely short event. In terms of the history of impact force, the maximum values were achieved in a time of less than 0.2 ms. Higher peaks are generally observed for thicker glasses. It is due to the fact that the kinetic energy applied to the impactor depends on the glass thickness and results from experiments, where the ascending trend was identified (Figure 3). The same observation applies to the principal stress in the glass, where peak values are identified within the first 0.5 ms of the simulation, thus slightly later than reaching the maximum value of the impact force. It is worth noting the general trend, in which the time at the maximum stress decreases for thicker samples. Figure 6 presents principal tensile stress maps throughout the analysis of the 10 mm specimen. 

Table 4 summarises the results of the simulations. It presents the maximum impact force values and principal tensile stress in glass for different thicknesses of glass samples found in Figure 5a,b. As can be seen from Table 4, the maximum impact force depends on the thickness of the glass, however, the same cannot be stated about the maximum stresses. In the simulations, the unfavourable stress resulting from the self-weight of the samples were neglected; therefore, the approach used in the analysis is conservative. In Table 4, the load duration factors *k_mod_* are given. It is expressed as a ratio of the maximum stress achieved in the simulation to the characteristic tensile bending strength of the toughened glass loaded in a quasi-static manner that equals 120 MPa according to [16]. From the analysis, the mean value of maximum principal stress in glass for all glass thicknesses is 168.42 ± 14.41 MPa which corresponds to the mean value of load duration factor *k_mod_* of 1.40 ± 0.12. 

The results of the current study show a good correlation with the reference research data. In terms of the load duration factor for toughened glass, the following values were reported by other researchers: 1.17–1.25 [33], 1.5–2.08 [34], 1.5 [35] and 1.08–1.15 [36] and 1.12 [29]. It should be noted that the latter was based on corundum-treated surface conditions, which could lead to a decrease of the bending strength. 

## 4. Case Study

The main reason to include a case study in the paper was to demonstrate the use of the modelling methodology and the results of the work on a practical example. The analysis aimed to determine the level of effort of the sample during impacts and show the potential capabilities of using numerical methods for assessing the load-bearing capacity of glass systems. 

### 4.1. Experimental Methodology

Experiments presented in this section were performed at the Building Research Institute ITB in Warsaw (Poland) as a standard test requested by an industrial client. The main purpose of this work was to evaluate the resistance of a monolithic glass partition wall to damage and functional failure from horizontal loads. 

The tests were performed on a sample with dimensions of 1343 × 2121 mm^2^ and 6 mm in thickness according to EAD 210005-00-0505 “Internal partition kits for use as non-load bearing walls” [65] that replaced ETAG 003 “Guideline for European technical approval for internal partition kits for use as non-loadbearing walls” [66].

**Figure 7:**
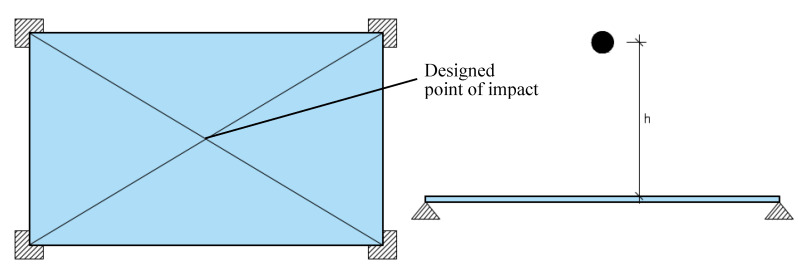
Assembly for hard body impact test based on 65.

The tests were conducted with two impactors (steel balls 50 and 63.5 mm in diameter) with the corresponding mass of 514 g and 1030 g, respectively. Before the experiments, the sample was horizontally positioned on four supports at the corners to allow the possibility of the impactor going through the panel in case of an unfavourable test result, thus ensuring the experiment’s safety (Figure 7). For the impact location, the most onerous point (centre of the panel) was chosen. In the test, the impactors were dropped from heights that resulted in the total impact energy according to [65]. 

### 4.2. Results

#### 4.2.1. Experimental Verification

Table 5 presents an overview and results of the performed experiments. The sample was hit several times with impactors released from different drop heights and corresponding impact energies. 

During the experiments, for all cases, no failure of glass occurred; thus, the sample passed the verification, and its resistance to damage and functional failure from horizontal loads was officially confirmed.

#### 4.2.2. Numerical Modelling

**Figure 8:**
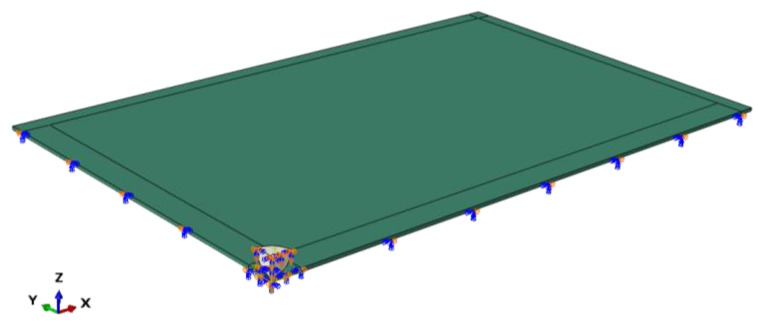
General view of FE numerical model of the case study (ABAQUS/Standard).

The sample was investigated numerically in terms of the level of effort for given impacts. A numerical model of the experimental set-up was developed using the commercial FE analysis software ABAQUS [52], see Figure 8. The modelling approach and all details regarding the FE model, material parameters, mesh etc., were the same as in the study presented in Section 3.2. 

**Figure 9:**
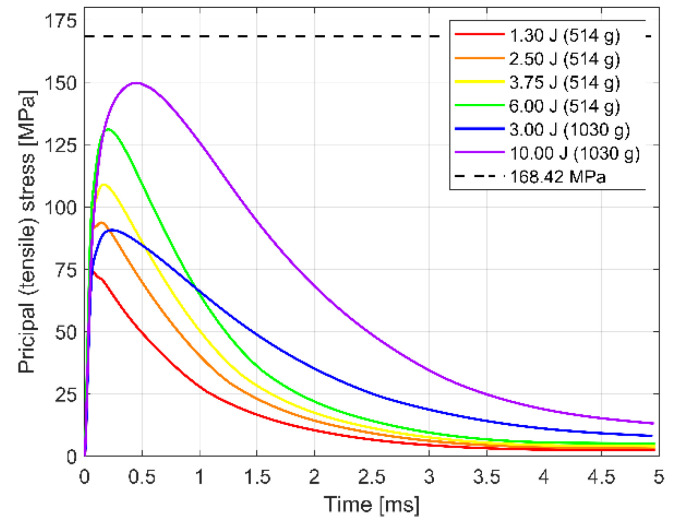
Results of numerical study: history of principal (tensile) stress in glass at the impact location.

Figure 9 presents the history of principal tensile stress in glass at impact location obtained from the numerical study. It is clear that the value of the stress depends on the impact energy; the larger impact energy produces higher stress values. In addition, the shape of the curves is strictly dependent on the mass of the impactor. For all cases, the maximum value of the principal tensile stress in the glass did not reach the value of the characteristic strength of FT glass for impact loads.

Table 6 summarises the results obtained from the numerical study. It reports the maximum values of principal tensile stress in glass at impact location for given impact energy and corresponding velocity of the impactor. In Table 6, the level of effort for all cases is also shown. It is defined as a ratio (in percentage) of the maximum stress achieved in the simulations to the characteristic impact strength of FT glass determined in this study. For the maximum value of the impact energy (10 J) with the 1030 g impactor, the maximum stress obtained is equal to 149.70 MPa, which results in an 88.9% level of effort. 

## 5. Conclusions

In this paper, based on the results of an experimental campaign, a non-linear 3D finite element model was developed to simulate the dynamic response of monolithic toughened glass panes subjected to hard-body impact. The model was used to determine retrospectively the impact strength of glass based on the maximum value of principal (tensile) stress in glass achieved for a given characteristic kinetic energy of the impactor determined in the physical experiments.

The experimental and numerical study found that the mean value of the maximum principal stress in glass for impact loads for FT glass calculated for all considered thicknesses equals 168.42 MPa. This results in the load duration factor of 1.40 for impact loads for FT glass compared to the characteristic quasi-static glass strength (120 MPa). The results of the current study show a good correlation with the available reference research data: 1.17–1.25 [33], 1.5–2.08 [34], 1.5 [35] and 1.08–1.15 [36] and 1.12 [29]. Moreover, a case study was presented to demonstrate the use of the modelling methodology and the results of the approach on a practical example. 

The current study shows the potential of numerical methods to investigate the dynamic behaviour of glass samples subjected to hard-body impacts. However, it should be noted that only the critical drop height causing glass fracture in the experimental campaign was reported. In this way, no additional data was collected to be used for the validation of the numerical model. Therefore, to obtain more research data, additional experiments are planned in the near future involving the use of high-resolution sensors. In addition, different boundary conditions of supports for glass plate and parameters such as level of fixity and continuity of supports at perimeters are planned to be investigated.

## Figures and Tables

**Table 1:** Rayleigh parameters for glass samples.

Glass Thickness [mm]	α	β	T_1_ [ms]	T_2_ [ms]	ζ [%]
5	4.16	2.08 × 10^−5^	20.62	9.54	1.0 [22,56]
6	4.99	1.73 × 10^−5^	17.18	7.97	
8	6.65	1.30 × 10^−5^	12.90	6.00	
10	8.30	1.05 × 10^−5^	10.32	4.82	
12	9.94	8.74 × 10^−5^	8.61	4.03	
15	12.39	7.03 × 10^−5^	6.89	3.25	

**Table 2:** Results of the experimental campaign.

Nominal Thickness [mm]	Number of Test	Impactor’s Weight [kg]	Mean Destructive Height [m]	Characteristic kinetic Energy E_k,k_ [J]
5	10	4.11	0.56 ± 0.19	12.71
6	35	4.11	0.76 ± 0.24	17.86
8	35	4.11	0.91 ± 0.29	21.20
10	35	4.11	1.37 ± 0.39	35.16
12	35	4.11	1.95 ± 0.54	49.28
15	35	4.11	2.11 ± 0.65	48.98

**Table 3:** Loads used in the numerical study.

Nominal Thickness [mm]	Impactor’s Weight [kg]	Characteristic kinetic Energy E_k,k_ [J]	Characteristic Velocity v_k_ [m/s]
5	4.11	12.71	2.49
6		17.86	2.95
8		21.20	3.21
10		35.16	4.14
12		49.28	4.90
15		48,98	4.88

**Table 4:** Results of the numerical study.

Glass Thickness [mm]	Max. Impact Force [N]	Max. Principal (Tensile) Stress [MPa]	*k_mod_*	Mean Stress [MPa]	Mean *k_mod_*
5	638.31	151.63	1.26	168.42 ± 14.41	1.40 ± 0.12
6	958.77	163.36	1.36		
8	1533.21	152.94	1.27		
10	3010.56	178.21	1.49		
12	4999.62	192.76	1.61		
15	7409.48	171.58	1.43		

**Table 5:** Details of the performed tests.

Impact Number	Impactor	Drop Height [m]	Impact Energy [J]	Verification
1	514 g	0.26	1.30	Passed
2		0.50	2.50	Passed
3		0.74	3.75	Passed
4		1.19	6.00	Passed
5	1030 g	0.30	3.00	Passed
6		0.99	10.00	Passed

**Table 6:** Results of the numerical study.

Impact Number	Impactor	Impact Energy [J]	Impactor’s Velocity [m/s^2^]	Max. Principal (Tensile) Stress [MPa]	Level of Effort [%]
1	514 g	1.30	2.25	74.01	43.9
2		2.50	3.12	83.68	55.6
3		3.75	3.82	108.97	64.7
4		6.00	4.83	131.03	77.8
5	1030 g	3.00	2.41	90.65	53.8
6		10.00	4.41	149.70	88.9

## Data Availability

Data available on request.
